# DNMT1高表达与beta-catenin蓄积及肺鳞癌、腺癌的恶性表型相关

**DOI:** 10.3779/j.issn.1009-3419.2010.09.04

**Published:** 2010-09-20

**Authors:** 洪涛 徐, 佐周 王, 娣 刘, 庆昌 李, 顺东 戴, 恩华 王

**Affiliations:** 110001 沈阳，中国医科大学基础医学院病理学教研室 Department of Pathology, College of Basic Medical Sciences, China Medical University, Shenyang 110001, China

**Keywords:** 肺肿瘤, DNMT1, β-catenin, Lung neoplasms, DNA methyltransferase 1, β-catenin

## Abstract

**背景与目的:**

DNA甲基转移酶1(DNA methyltransferase 1, DNMT1)是调控DNA甲基化的重要分子之一，DNMT1的异常表达与抑癌基因的甲基化、失活和多种肿瘤的发生发展有关。本研究旨在分析阐明DNMT1在正常肺组织和肺癌组织中表达的差异及其与肺鳞癌和腺癌临床病理因素的关系，并探讨DNMT1与β-catenin在肺癌中表达的相关性。

**方法:**

采用组织芯片和免疫组化方法检测DNMT1和β-catenin在84例肺鳞癌、腺癌和相应癌旁正常肺组织中的表达情况。

**结果:**

DNMT1在84例肺癌组织中的平均阳性率为(58.04±35.07) %，显著高于癌旁正常肺组织[(6.88±10.26) %](*t*=12.835, *P*＜0.001)。DNMT1的高表达与肺癌组织的腺癌组织学分型(*r*=0.365, *P*=0.001)、低分化程度(*r*=0.253, *P*=0.021)和淋巴结转移(*r*=0.246, *P*=0.024)正相关。DNMT1与β-catenin的细胞浆表达显著正相关(*r*=0.571, *P*＜0.001)。

**结论:**

DNMT1的高表达是肺鳞癌和腺癌的普遍现象，DNMT1的高表达与肺癌的腺癌组织学类型和恶性表型有关；DNMT1在肺癌中可能与β-catenin协同表达。

## Abstract (en)

**Background and objective:**

DNA methyltransferase 1 (DNMT1) is one of the important molecules regulating DNA methylation. The abnormal expression of DNMT1 was associated with the methylation and inactivation of tumor suppressor gene and tumorigenesis. The aim of this study is to clarify the difference of DNMT1 expression between lung cancer tissues and corresponding normal lung tissues, to analyze the relationships between DNMT1 expression and clinicopathologic characteristics of lung squamous cell carcinoma and adenocarcinoma, and to investigate the correlation between the expressions of DNMT1 and β-catenin.

**Methods:**

The expressions of DNMT1 and β-catenin were examined in 84 lung squamous cell carcinoma and adenocarcinoma tissues and corresponding normal lung tissues using tissue microarray and immunohistochemistry.

**Results:**

The average positive rate of DNMT1 was significantly higher in 84 lung cancer tissues [(58.04 ±35.07)%] than that in corresponding normal lung tissues [(6.88±10.26)%](*t*=12.835, *P* < 0.001). The high expression of DNMT1 was positively correlated with adenocarcinoma histological type (*r*=0.365, *P*=0.001), poor differentiation (*r*=0.253, *P*=0.021) and lymph node metastasis (*r*=0.246, *P*=0.024) in lung cancer. The expression of DNMT1 was significantly correlated with the cytoplasmic expression of β-catenin (*r*=0.571, *P* < 0.001).

**Conclusion:**

The high expression of DNMT1 was a common phenomenon in lung squamous cell carcinoma and adenocarcinoma. The high expression of DNMT1 was correlated with the malignant phynotype of lung cancer. DNMT1 may express coordinately with β-catenin in lung cancer.

DNA甲基化是导致抑癌基因失活和肿瘤发生发展的重要表观遗传学因素，在肺癌中已发现*CDH1* (E-cadherin)、*p16*、*p53*、*PTEN*、*RB*等多种抑癌基因的甲基化^[1]^。DNA甲基转移酶1(DNA methyltransferase 1, DNMT1)是调控CpG二核苷酸中胞嘧啶甲基化的重要分子之一，研究表明，DNMT1的异常表达与结肠癌^[2]^、乳腺癌^[3]^、胰腺癌^[4]^等多种恶性肿瘤的发生发展有关。但目前有关DNMT1在肺癌中表达和作用的研究尚处于起步阶段，DNMT1在肺鳞癌、腺癌中的表达情况和意义尚不明确。β-连环蛋白(β-catenin)作为细胞粘附和Wnt通路的关键分子，与肺癌的发生发展和侵袭转移关系密切^[5]^。而DNMT1与β-catenin在肺癌中是否存在某些联系和相互作用尚不明确。为此，本研究采用组织芯片和免疫组化方法检测DNMT1在肺鳞癌、腺癌和癌旁正常肺组织中的表达情况，分析阐明DNMT1在正常肺组织和肺癌组织中表达的差异及其与肺鳞癌和腺癌临床病理因素的关系，并探讨DNMT1与β-catenin在肺癌中表达的相关性。

## 1 材料与方法

### 1.1 材料

#### 1.1.1 病例与标本

84例肺鳞癌、腺癌和相对应的84例癌旁正常肺组织标本取自上海芯超生物科技有限公司组织库存档标本(由医院合作方提供)，癌旁正常肺组织的取材部位距离癌灶1 cm-2 cm，由上海芯超公司的病理专家根据WHO(2004年)肺肿瘤组织学分类标准复诊确认并制备组织芯片(阵列编号：OD-CT-RsLug01-002)，每例标本直径1.0 mm。根据存档资料，患者平均年龄(61.21±11.74)岁(20岁-81岁)(3例年龄信息缺失)，男性49例，女性33例(2例性别信息缺失)，术前均未接受放化疗。肺癌组织根据WHO(2004年)肺肿瘤组织学分类标准分为鳞癌37例(包括高分化14例、中分化14例、低分化9例)；腺癌47例(包括高分化12例、中分化27例、低分化8例)，75例肿瘤最大直径≥3 cm，35例存在淋巴结转移。

#### 1.1.2 试剂

鼠抗人DNMT1单克隆抗体(sc-70981)购自美国Santa Cruz生物技术公司，鼠抗人β-catenin单克隆抗体(610153)购自美国BD Transduction Laboratories，SP超敏免疫组化试剂盒(KIT-9710)和DAB酶底物显色试剂盒(DAB-0031)均购自福州迈新生物技术公司。

### 1.2 方法

将组织芯片标签向上并倾斜一定角度，置于60 ℃烤箱中3 h，融掉表面封蜡，经脱蜡、脱苯、水化后，3%H_2_O_2_封闭内源性过氧化物酶15 min，0.1%枸橼酸缓冲液(pH 6.0-6.2)中高温高压修复1.5 min，山羊血清37 ℃封闭20 min，滴加鼠抗人DNMT1一抗(1:200)或鼠抗人β-catenin一抗(1:200)，4 ℃过夜后，依次加入生物素标记二抗和链霉素抗生物素蛋白-过氧化酶，分别于37 ℃下孵育30 min，DAB显色后苏木素复染，梯度酒精脱水，二甲苯透明，中性树胶封片。以组织芯片中癌旁正常肺组织作为内对照，以PBS取代一抗作为阴性对照。

结果判定：免疫组化染色后，组织芯片中组织形态结构较完好、能够进行细胞染色阳性判定和计数的标本为有效病例。芯片中每一例组织标本直径为1.0 mm，所以能够在显微镜下观察并计数每一例有效病例的瘤细胞总数和阳性瘤细胞数，得出每例标本DNMT1和β-catenin表达的阳性率，并计算出DNMT1在肺癌组织中的平均阳性率。删除因掉片脱点而无法计数的病例。本研究中，DNMT1在肺癌组织中的平均阳性率为(58.04±35.07) % (*n*=84)，所以将阳性率≥58%定为DNMT1高表达，阳性率＜58%或阴性定为DNMT1低表达。

### 1.3 统计学分析

运用SPSS 13.0进行数据分析，采用独立样本*t*检验分析DNMT1的表达在肺癌组织和癌旁正常肺组织间的差异；采用卡方检验、似然比法和*Spearman*等级相关分析DNMT1的表达与肺鳞癌和腺癌临床病理因素的关系；采用*Pearson*相关分析DNMT与β-catenin表达的关系。*P*＜0.05为差异有统计学意义。

## 2 结果

### 2.1 DNMT1在肺鳞癌和腺癌中的表达显著高于癌旁正常肺组织

DNMT1免疫组化染色后，详细计数每一例肺癌和癌旁正常肺组织中DNMT1的阳性表达细胞数，并计算出阳性表达率。结果发现，84例癌旁正常肺组织中，有43例出现DNMT1在少数上皮细胞浆中的微弱表达，平均阳性表达率为(6.88±10.26) %(*n*=84)，而在84例肺鳞癌和腺癌组织中，75例(89.29%)存在不同程度的DNMT1细胞浆(伴或不伴有细胞核)表达，平均阳性表达率为(58.04±35.07) %(*n*=84)，显著高于癌旁正常肺组织(*t*=12.835, *P*＜0.001)(图 1，表 1)。

**1:**
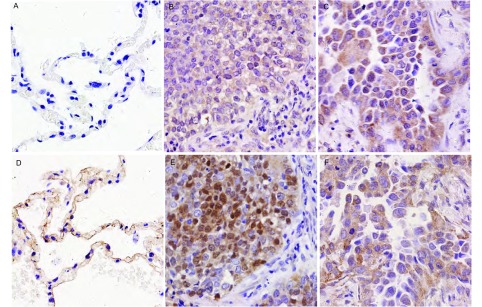
DNMT1和*β*-catenin在肺鳞癌、腺癌和癌旁正常肺组织中的表达(×400)。A：DNMT1在癌旁正常肺组织中阴性表达；B、C：DNMT1在肺鳞癌(B)和肺腺癌(C)中的细胞浆和细胞核表达；D：*β*-catenin在癌旁正常肺组织中的膜表达；E：*β*-catenin在肺鳞癌中的细胞浆和细胞核表达；F：*β*-catenin在肺腺癌中的细胞浆表达。 The expressions of DNMT1 and *β*-catenin in lung squamous cell carcinoma, adenocarcinoma and corresponding normal lung tissues (×400). A: The negative expression of DNMT1 in corresponding normal lung tissue; B, C: The cytoplasmic-nuclear expression of DNMT1 in lung squamous cell carcinoma (B) and adenocarcinoma (C); D: The membranous expression of *β*-catenin in corresponding normal lung tissue; E: The cytoplasmicnuclear expression of *β*-catenin in lung squamous cell carcinoma; F: The cytoplasmic expression of *β*-catenin in lung adenocarcinoma.

**1:** DNMT1在肺癌和癌旁正常肺组织中的表达 The expression of DNMT1 in lung cancer and corresponding normal lung tissues

Group	*n*	Expression of DNMT1			
		Mean expression rate	Standard division	*t*	*P*
Lung cancer tissue	84	58.04%	35.07%	12.835	＜0.001
Corresponding normal lung tissue	84	6.88%	10.26%		

### 2.2 DNMT1的高表达与肺鳞癌和腺癌的分型、分化程度和淋巴结转移相关

根据DNMT1高、低表达的判定标准，肺癌中有53例(63.10%)为DNMT1高表达，31例(36.90%)为DNMT1低表达。如表 2所示，DNMT1在肺腺癌中的高表达率(37/47, 78.72%)显著高于肺鳞癌(16/37, 43.24%)(*P*=0.001)；在中低分化肺癌中的高表达率(41/58, 70.69%)显著高于高分化肺癌(12/26, 46.15%)(*P*=0.021)；在有淋巴结转移的病例中的高表达率(27/35, 77.14%)显著高于无淋巴结转移的病例(26/49, 53.06%)(*P*=0.024)，而且DNMT1的表达在不同性别(*P*=0.018)、不同年龄段(*P*=0.023)的病例间也有统计学差异。但本研究未发现DNMT1的表达与肿瘤大小之间的关系(*P*=0.623)。进一步的Spearman等级相关分析也表明DNMT1的高表达与肺癌的组织学类型(*r*=0.365, *P*=0.001)、分化程度(*r*=0.253, *P*=0.021)、淋巴结转移(*r*=0.246, *P*=0.024)、性别(*r*=0.262, *P*=0.017)和年龄(*r*=-0.253, *P*=0.022)相关。

**2:** DNMT1的表达与肺鳞癌和腺癌临床病理因素的关系 Relationships between DNMT1 expression and clinicopathologic characteristics of lung squamous carcinoma and adenocarcinoma

Characteristics	*n*	Expression of DNMT1					
		High expression (*n*)	High expression rate	*χ*^2^	*P*	*r*	*P*
Gender				5.626	0.018	0.262	0.017
Male	49	26	53.06%				
Female	33	26	78.79%				
Age				5.199	0.023	-0.253	0.022
＜61	36	28	77.78%				
≥61	45	24	53.33%				
Histological type				11.192	0.001	0.365	0.001
Squamous cell carcinoma	37	16	43.24%				
Adenocarcinoma	47	37	78.72%				
Differentiation				4.641	0.031	0.235	0.031
Well	26	12	46.15%				
Moderate-poor	58	41	70.69%				
Tumor size				0.241	0.623	0.054	0.625
＜3 cm	9	5	55.56%				
≥3 cm	75	48	64.00%				
Lymph node metastasis				5.085	0.024	0246	0.024
Yes	35	27	77.14%				
No	49	26	53.06%				

### 2.3 DNMT1的表达与β-catenin的细胞浆表达相关

β-catenin免疫组化染色后，组织芯片中获得有效病例77例。所有肺鳞癌和腺癌病例中均不同程度地出现了β-catenin的细胞浆表达，其平均表达率为(69.74±27.14) %，其中28例伴有不同程度的细胞膜表达(36.37%)，7例伴有细胞核表达(9.09%)。β-catenin的细胞浆表达与肺癌的组织分型(*r*=0.301, *P*＜0.008)、肿瘤大小(*r*=0.204, *P*＜0.037)和淋巴结转移(*r*=0.278, *P*＜0.014)相关，但未发现β-catenin细胞膜和细胞核表达与肺癌临床病理因素的相关性，因已有大量关于β-catenin在肺癌中表达和意义的报道^[5-7]^，为避免重复，本研究对β-catenin与肺癌临床病理因素的相关性不再赘述。经*Pearson*相关分析后发现DNMT1与β-catenin的细胞浆表达显著相关(*r*=0.571, *P*＜0.001)(图 1)，而与β-catenin的细胞膜(*r*=0.117, *P*=0.311)和细胞核表达(*r*=0.017, *P*=0.885)无明显相关性。

## 3 讨论

肺癌是目前世界上最常见的恶性肿瘤之一，肺癌患者的死亡率在工业化国家中居恶性肿瘤之首^[8]^。抑癌基因的甲基化是肺癌发生和发展的重要原因之一，大量研究^[1]^表明肺癌中经常出现*CDH1*、*p16*、*p53*、*PTEN*、*RB*、*APC*^[9]^等多种抑癌基因的甲基化和失活。

DNA甲基转移酶是调控CpG二核苷酸中胞嘧啶甲基化的关键分子，分为DNMT1、DNMT2和DNMT3三个亚家族，DNMT3又可分为DNMT3A和DNMT3B，它们不仅对维持染色体结构的稳定性、保障基因组表观遗传信息在亲代细胞间的正确传递有重要作用，还与结肠癌^[2]^、乳腺癌^[3]^、胰腺癌^[4]^、肺癌^[10]^等多种恶性肿瘤中抑癌基因的甲基化、失活和肿瘤的发生发展、侵袭转移有关。目前研究^[1]^表明DNMT1是在肺癌中起主要作用的DNA甲基转移酶亚型。

为深入探讨DNMT1与肺癌发生发展的关系，本研究首先检测了DNMT1在肺癌和癌旁正常肺组织中表达的差异，结果发现，在84例癌旁正常肺组织中，只有约半数的肺组织(43例)中出现微量的DNMT1表达，平均表达率仅为(6.88±10.26) %，说明在正常肺组织中DNMT1处于低水平状态，只有少数上皮细胞表达。而肺癌组织中，有89.29%的病例出现DNMT1不同程度的表达，平均表达率为(58.04±35.07) %，显著高于癌旁正常肺组织(*P*＜0.001)，说明DNMT1的高表达在肺癌中是普遍现象，这种大大超过正常水平的DNMT1的表达量，可能是导致大量抑癌基因甲基化、失活和肺癌形成、发展并侵袭转移的重要原因之一。那么，DNMT1的高表达与肺癌发生发展和侵袭转移的具体关系如何呢？为此，本研究详细分析了DNMT1与肺癌临床病理因素的关系，结果发现DNMT1的高表达与肺癌的组织学分型、分化程度和淋巴结转移均有显著的相关性，提示DNMT1的表达情况有可能为判断肿瘤类型、恶性程度和预后提供重要的信息。与本研究结果相似，在结肠癌^[2]^、胰腺癌^[4]^、肝细胞癌^[11]^和粘液表皮样癌^[12]^等肿瘤中均发现DNMT1的表达水平有明显升高，并不同程度地与肿瘤的分化程度、临床分期或(和)预后不良相关。Lin等^[10]^发现DNMT1在肺癌中的表达显著，并与吸烟患者的预后不良相关，进一步的研究^[13]^表明吸烟将导致DNMT1降解失调并在细胞浆、核内蓄积，进而诱导抑癌基因甲基化和肿瘤的形成。本研究发现DNMT1的高表达与女性患者、肺腺癌相关，与此相似，Lin等^[14]^发现DNMT3b的高表达更常出现在腺癌组织和女性患者中，这可能与雌激素水平或腺癌病例中女性比例较高有关，其原因有待进一步研究证实。

β-catenin是Wnt通路的关键分子和重要的粘附分子，在正常上皮组织中定位于细胞膜，而在肿瘤中常出现异常的细胞浆和细胞核蓄积，并与Wnt通路的活化以及c-myc、Cyclin D1等靶基因的转录有关，以往的研究^[5-7, 15]^表明β-catenin异常蓄积与肺癌的恶性表型和预后不良密切相关。本研究首次发现DNMT1与β-catenin的细胞浆表达呈显著正相关，这表明它们二者之间存在着直接或间接的相互协同作用。有研究^[9, 16]^表明，肿瘤中存在着Wnt通路负调控基因的甲基化，DNMT1可能通过*Axin*、*APC*等基因的甲基化，促进了β-catenin的失调控和蓄积，另外，β-catenin也可能通过多种靶基因产物直接或间接地促进DNMT1的表达，二者可能具有相互调控、相互协同，共同促进肺癌发生发展和侵袭转移的作用。这一现象为深入研究肺癌中DNMT1与Wnt通路的调控机制开拓了新的方向。

综上所述，DNMT1的高表达是肺鳞癌和腺癌的普遍现象；DNMT1的高表达与肺癌的腺癌组织学类型、低分化和淋巴结转移相关；DNMT1与β-catenin的细胞浆表达呈显著正相关。深入研究DNMT1在肺癌中的表达调控机制将有可能为肺癌的早期诊断和基因靶向治疗提供新的线索和思路。

## References

[1] Tang M, Xu W, Wang Q (2009). Potential of DNMT and its epigenetic regulation for lung cancer therapy. Curr Genomics.

[2] Foran E, Garrity-Park MM, Mureau C (2010). Upregulation of DNA methyltransferase-mediated gene silencing, anchorage-independent growth, and migration of colon cancer cells by interleukin-6. Mol Cancer Res.

[3] Kastl L, Brown I, Schofield AC (2010). Altered DNA methylation is associated with docetaxel resistance in human breast cancer cells. Int J Oncol.

[4] Wang W, Gao J, Man XH (2009). Significance of DNA methyltransferase-1 and histone deacetylase-1 in pancreatic cancer. Oncol Rep.

[5] Königshoff M, Eickelberg O (2010). WNT signaling in lung disease: a failure or a regeneration signal?. Am J Respir Cell Mol Biol.

[6] Xu HT, Wang L, Lin D (2006). Abnormal beta-catenin and reduced axin expression are associated with poor differentiation and progression in nonsmall cell lung cancer. Am J Clin Pathol.

[7] Xu HT, Lin D, Wang L (2004). Expression and mutation of β-catenin in nonsmall cell lung cancer. Chin J Lung Cancer.

[8] Jemal A, Murray T, Ward E (2005). Cancer statistics. CA Cancer J Clin.

[9] Tsou JA, Shen LY, Siegmund KD (2005). Distinct DNA methylation profiles in malignant mesothelioma, lung adenocarcinoma, and non-tumor lung. Lung Cancer.

[10] Lin RK, Hsu HS, Chang JW (2007). Alteration of DNA methyltransferases contributes to 5'CpG methylation and poor prognosis in lung cancer. Lung Cancer.

[11] Fan H, Zhao ZJ, Cheng J (2009). Overexpression of DNA methyltransferase 1 and its biological significance in primary hepatocellular carcinoma. World J Gastroenterol.

[12] Shieh YS, Shiah SG, Jeng HH (2005). DNA methyltransferase 1 expression and promoter methylation of E-cadherin in mucoepidermoid carcinoma. Cancer.

[13] Lin RK, Hsieh YS, Lin P (2010). The tobacco-specific carcinogen NNK induces DNA methyltransferase 1 accumulation and tumor suppressor gene hypermethylation in mice and lung cancer patients. J Clin Invest.

[14] Lin TS, Lee H, Chen RA (2005). An association of DNMT3b protein expression with P16INK4a promoter hypermethylation in non-smoking female lung cancer with human papillomavirus infection. Cancer Lett.

[15] Pagaki E, Patsouris E, Gonidi M (2010). The value of E-cadherin/beta-catenin expression in imprints of NCSLC: relationship with clinicopathological factors. Diagn Cytopathol.

[16] Yeh KT, Chang JG, Lin TH (2003). Correlation between protein expression and epigenetic and mutation changes of Wnt pathway-related genes in oral cancer. Int J Oncol.

